# Molecular Insights into Human Placentation: From Villous Morphogenesis to Pathological Pathways and Translational Biomarkers

**DOI:** 10.3390/ijms26199483

**Published:** 2025-09-28

**Authors:** Ioana Vornic, Radu Caprariu, Dorin Novacescu, Alina Cristina Barb, Victor Buciu, Adelina Băloi, Diana Szekely, Cristian Silviu Suciu, Catalin Dumitru, Raul Patrascu, Flavia Zara, Cristina Stefania Dumitru

**Affiliations:** 1 Discipline of Gynecology, Department Medicine, Vasile Goldis Western University, Liviu Rebreanu Boulevard, No. 86, 310414 Arad, Romania; ioana_vornic@yahoo.com; 2 Discipline of Radiology and Medical Imaging, Department XV, Victor Babes University of Medicine and Pharmacy Timisoara, E. Murgu Square, Nr. 2, 300041 Timisoara, Romania; 3 Department II of Microscopic Morphology, Discipline of Histology, Victor Babes University of Medicine and Pharmacy Timisoara, E. Murgu Square, No. 2, 300041 Timisoara, Romania; toma.alina@umft.ro (A.C.B.); cristian_suciu@umft.ro (C.S.S.); flavia.zara@umft.ro (F.Z.); cristina-stefania.dumitru@umft.ro (C.S.D.); 4 Doctoral School, Victor Babes University of Medicine and Pharmacy Timisoara, E. Murgu Square, No. 2, 300041 Timisoara, Romania; victor.buciu@umft.ro (V.B.); diana.szekely@umft.ro (D.S.); 5 Department X–Surgery II, Disclipline Anaesthesia and Intensive Care, Victor Babes University of Medicine and Pharmacy Timisoara, E. Murgu Square, Nr. 2, 300041 Timisoara, Romania; adelina.baloi@umft.ro; 6 Department of Obstetrics and Gynecology, Victor Babes University of Medicine and Pharmacy Timisoara, Eftimie Murgu Square 2, 300041 Timisoara, Romania; dumitru.catalin@umft.ro; 7 Department of Functional Sciences, Victor Babes University of Medicine and Pharmacy Timisoara, 300041 Timisoara, Romania; patrascu.raul@umft.ro

**Keywords:** placenta, molecular insights, trophoblast invasion, maternal–fetal interface, preeclampsia, fetal growth restriction, placenta accreta spectrum, biomarkers, multi-omics

## Abstract

Placental dysfunction underlies the major obstetric syndromes, including preeclampsia, fetal growth restriction, placenta accreta spectrum, pregnancy loss, and monochorionic twin complications. Recent molecular studies have revealed that dysregulated oxygen sensing, impaired angiogenic signaling, altered immune tolerance, and defective trophoblast fusion represent shared pathogenic pathways that converge across these disorders. Integrating morphological evidence with mechanistic data highlights how villous maldevelopment, shallow trophoblast invasion, and aberrant vascular remodeling translate into clinical disease. Advances in biomarker research have already transformed clinical care: the sFlt-1/PlGF ratio is now established in the prediction and management of preeclampsia, while placental proteins such as PAPP-A and PP13, nucleic acid signatures including cfDNA, cfRNA and miRNAs, and extracellular vesicle cargo show promising potential for early, non-invasive detection of placental pathology. Multi-omics approaches, particularly single-cell and spatial transcriptomics combined with proteomic and metabolomic profiling, are paving the way for composite diagnostic panels that capture the polygenic and multicellular nature of placental disease. This review synthesizes current knowledge of molecular mechanisms, histological correlates, and translational biomarkers, and outlines how precision obstetrics may emerge from bridging mechanistic discoveries with clinical applications.

## 1. Introduction

The human placenta is a transient but indispensable organ that orchestrates maternal–fetal interactions, ensuring fetal growth, immune tolerance, and hormonal regulation throughout gestation. Derived from the trophectoderm, it establishes direct contact with maternal blood via its villous tree, a structure that expands and remodels dynamically to accommodate the increasing metabolic demands of the developing fetus [1]. Placental health is therefore critical for pregnancy success, with defective placentation representing a central mechanism underlying the “great obstetrical syndromes,” including preeclampsia (PE), intrauterine growth restriction (IUGR), placenta accreta spectrum (PAS), preterm birth, and stillbirth [2,3].

A growing body of evidence highlights that the development and pathology of the placenta are tightly governed by molecular and cellular pathways. Extravillous trophoblasts (EVTs) invade maternal spiral arteries, replacing the endothelium and transforming them into high-capacitance, low-resistance vessels; failure of this process results in shallow placentation and maternal vascular malperfusion [4,5]. Syncytiotrophoblasts, generated through cytotrophoblast fusion mediated by GCM1 and Syncytin proteins, form the outermost exchange interface and secrete key hormones such as Human chorionic gonadotropin (hCG) and human placental lactogen [6,7]. These processes are influenced by oxygen gradients, with hypoxia-inducible factors (HIFs) and vascular endothelial growth factor (VEGF) signaling orchestrating trophoblast proliferation, differentiation, and angiogenesis in early gestation [8]. In parallel, maternal immune cells, particularly uterine natural killer (uNK) cells, interact with EVTs via KIR/HLA-C signaling to fine-tune invasion and vascular remodeling [9]. Dysregulation of these pathways is implicated in pregnancy complications, emphasizing the need for an integrated understanding of placental morphogenesis and molecular regulation.

Beyond its developmental biology, the placenta has emerged as a critical source of clinically actionable biomarkers. Circulating factors such as soluble fms-like tyrosine kinase-1 (sFlt-1) and placental growth factor (PlGF) are now validated tools for predicting and managing preeclampsia [10,11]. Other molecules, including pregnancy-associated plasma protein-A (PAPP-A), placental protein 13 (PP13), microRNAs, and extracellular vesicles, are being investigated as early indicators of placental dysfunction [12,13]. The rapid adoption of the soluble fms-like tyrosine kinase-1/placental growth factor (sFlt-1/PlGF) ratio in clinical practice illustrates how mechanistic insights into placental angiogenesis can translate into diagnostic innovation with direct impact on maternal–fetal care. However, many other promising candidates remain under validation, underscoring a gap between discovery and clinical implementation.

Recent technological advances have revolutionized our ability to interrogate placental biology. Single-cell RNA sequencing and spatial multi-omics have mapped the cellular diversity and spatial organization of trophoblast, immune, and stromal populations at unprecedented resolution [13]. Organoid systems and placenta-on-a-chip models now provide physiologically relevant platforms to study implantation, trophoblast differentiation, and drug responses ex vivo [14,15]. As a concrete exemplar, microengineered placenta-on-a-chip platforms can reproduce maternal–fetal barrier functions and reveal altered nutrient/drug transfer under hypoxic stress; however, current devices remain constrained by simplified cell composition and short culture durations [14,15]. These experimental models, coupled with omics-based biomarker discovery, represent the forefront of placental research and hold potential to identify novel diagnostic and therapeutic strategies.

Despite these advances, significant knowledge gaps persist. The precise timing and molecular “switches” that regulate the transition from histiotrophic to hemotrophic nutrition remain incompletely understood. The bidirectional dialogue between maternal immune tolerance and trophoblast invasion, though increasingly illuminated, is still not fully deciphered. Moreover, the clinical translation of molecular biomarkers and therapeutic strategies is hindered by inter-individual variability and the heterogeneity of placental pathologies [16,17]. Bridging these gaps requires a synthesis of classical histological perspectives with cutting-edge molecular insights.

This review aims to provide an integrative overview of placental development and pathology, focusing on the molecular pathways that govern villous morphogenesis, trophoblast invasion, and syncytialization. We highlight how dysregulation of these mechanisms contributes to pregnancy complications and discuss emerging diagnostic biomarkers and experimental models that hold promise for clinical translation. By combining morphological, molecular, and clinical perspectives, this article seeks to advance a holistic understanding of human placentation and to outline future directions in placental pathology and diagnostics. It is designed as a comprehensive narrative review, synthesizing current evidence from histology, molecular biology, and clinical studies, with an emphasis on translational insights.

*Review Methods*. This narrative review was based on a structured literature search conducted in PubMed, Scopus, and Web of Science databases, covering publications up to July 2025. Search terms included combinations of “placenta,” “trophoblast,” “villous morphogenesis,” “angiogenesis,” “immune tolerance,” “preeclampsia,” “fetal growth restriction,” “placenta accreta spectrum,” “pregnancy loss,” “twin-to-twin transfusion syndrome,” and “biomarkers.” Only peer-reviewed original studies, systematic reviews, and meta-analyses published in English were included; conference abstracts, editorials, and non-peer-reviewed sources were excluded. Priority was given to prospective cohort studies, large case–control series, and systematic reviews/meta-analyses, while narrative reviews and smaller case reports were used selectively to provide contextual background. The evidence was synthesized thematically, emphasizing the progression from molecular mechanisms to histological correlates and clinical applications.

## 2. Molecular and Cellular Basis of Normal Placentation

Placental development begins at the blastocyst stage when the trophectoderm differentiates into cytotrophoblasts (CTBs) and syncytiotrophoblasts (STBs), and the conceptus engages the receptive endometrium. A highly regulated sequence of cell fate decisions and environmental cues—including oxygen gradients, immune tolerance, and angiogenic signaling—controls implantation, villous morphogenesis, extravillous trophoblast (EVT) invasion, and the shift from histiotrophic to hemotrophic nutrition. Recent single-cell RNA sequencing (scRNA-seq) and spatial multi-omics studies have provided unprecedented resolution of trophoblast subtypes, their trajectories, and interactions at the maternal–fetal interface, reshaping our understanding of placental development in both physiological and pathological contexts [18].

### 2.1. Blastocyst Implantation and Trophoblast Differentiation

By day 5 post-fertilization, the blastocyst reaches the uterine cavity, where it undergoes apposition, adhesion, and invasion into the decidualized endometrium. At this stage, trophoblast differentiation results in two major lineages: CTBs, which are mitotically active and maintain proliferative capacity, and STBs, a multinucleated layer formed by CTB fusion. This fusion is orchestrated by the transcription factor GCM1 and fusogenic retroviral-derived proteins such as Syncytin-1, with recent studies highlighting their regulation by cAMP signaling and epigenetic modulators. Invasion is supported by matrix metalloproteinases (MMP-2, MMP-9) and integrin switching that allow penetration of the endometrial stroma and remodeling of the extracellular matrix [19,20,21].

Early implantation occurs in a low-oxygen microenvironment (~20 mmHg), which stabilizes HIF-1α and maintains a proliferative CTB phenotype. This hypoxic niche favors angiogenic signaling through VEGF and PlGF, enabling the coordinated establishment of primitive uteroplacental circulation while protecting the embryo from oxidative stress. Recent molecular mapping has refined the timing of this oxygen transition, showing that premature loss of trophoblastic plugs can expose the placenta to oxidative stress and trigger early pregnancy loss [22,23].

### 2.2. Chorionic Villous Development

Chorionic villi emerge in a stepwise manner: primary villi (day 11–13) are composed of CTBs covered by STB; secondary villi (day 16–18) incorporate extraembryonic mesoderm into the villous core; and tertiary villi (from day 20 onward) contain fetal capillaries, connecting the developing placental vasculature to the embryonic circulation. This sequence culminates in a hemochorial placenta, where maternal blood directly bathes the villous surface, a defining feature of human placentation [19,24].

Villous maturation progresses through mesenchymal, immature intermediate, mature intermediate, stem, and terminal villi. Each type exhibits distinctive stromal and vascular characteristics that reflect developmental stage and functional specialization. Terminal villi, the smallest branches of the villous tree, form extensive vasculosyncytial membranes, in which STB cytoplasm, fused basement membranes, and fetal endothelial cells create a highly attenuated barrier that minimizes diffusion distance and optimizes maternal–fetal exchange [25].

**Figure 1:**
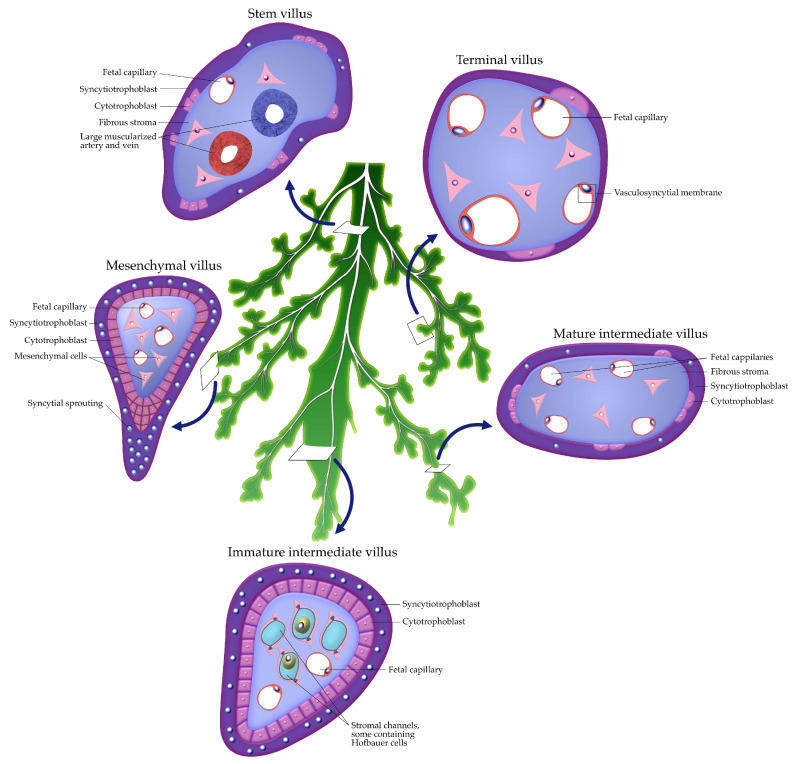
Schematic representation of placental villous subtypes. Stem villi form the main supporting trunk of the villous “tree,” harboring large vessels and connective tissue for structural support. From this trunk, the remaining villi represent successive stages of maturation arranged in a counterclockwise direction. Mesenchymal villi represent the earliest form, with abundant stromal cells and few capillaries. Immature intermediate villi show stromal channels and Hofbauer cells. Mature intermediate villi contain more developed fetal capillaries within a fibrous stroma. Terminal villi, with peripheralized capillaries and vasculosyncytial membranes, are specialized for efficient maternal–fetal exchange.

The structural hierarchy and histological features of villous subtypes are summarized in Figure 1, illustrating the transition from mesenchymal to terminal villi and their progressive specialization for maternal–fetal exchange.

Recent spatial transcriptomic and proteomic atlases demonstrate that villous branching and angiogenesis are driven by finely tuned VEGF/PlGF gradients, NOTCH signaling, and dynamic cross-talk among trophoblasts, Hofbauer cells, and fetal endothelial cells. These findings emphasize that villous morphogenesis is not only a structural process but also a molecularly orchestrated one, directly influencing the efficiency of gas and nutrient exchange [26,27].

### 2.3. Trophoblast Invasion and Spiral Artery Remodeling

Extravillous trophoblast (EVT) invasion drives the stepwise remodeling of uterine spiral arteries (SAR)—loss of maternal endothelium, degradation of the musculoelastic media, fibrinoid deposition, and luminal dilation—converting high-resistance vessels into high-capacity, low-resistance channels that sustain placental perfusion. Updated immunologic and systems reviews emphasize the coordinated roles of decidual stromal, endothelial, and immune cells in licensing this program and underscore the clinical impact of incomplete SAR on placental perfusion [28,29].

The uNK-EVT dialog (KIR/HLA axis). Efficient SAR depends on a tightly regulated uterine NK (uNK)-EVT cross-talk, in which EVT-expressed HLA-C/E/G engage maternal KIR receptors to shape uNK cytokine, chemokine, and protease output, thereby tuning EVT migration and vascular conversion. Contemporary immunogenetic and mechanistic syntheses link specific KIR-HLA-C combinations with preeclampsia and fetal growth restriction risk, supporting a causal role for perturbed uNK licensing in “shallow” placentation [30,31].

As detailed in Section 2.1, oxygen sensing via HIF signaling initially sustains trophoblast proliferation; its dysregulation later contributes to defective EVT differentiation and impaired spiral artery remodeling. Recent work refines this model: HIF signaling modulates EVT migration/invasion, and dysregulated HIF activity (or its upstream regulators) perturbs invasion and is implicated in hypertensive disorders of pregnancy [32,33].

Single-cell & spatial maps of SAR. Single-cell and spatial multi-omics atlases now resolve discrete EVT states (interstitial vs. endovascular), their gene-regulatory programs, and cell–cell circuits with decidual immune and stromal partners across gestation, providing a molecular blueprint for normal vs. insufficient remodeling. These datasets delineate micro-niches that sustain invasion, highlight angiogenic modules, and offer reference points for disease [34,35]. For example, spatial maps have resolved interstitial versus endovascular EVT states along the spiral-artery remodeling gradient, offering a mechanistic frame for insufficient remodeling in disease; nonetheless, atlases provide correlative snapshots and require orthogonal functional validation [34,35].

### 2.4. Syncytiotrophoblast Formation and Endocrine Functions

The STB, a multinucleated epithelial layer formed by fusion of CTBs, is the principal site for nutrient and gas exchange, as well as hormone production crucial for pregnancy maintenance. Recent mechanistic insights and experimental models have expanded our understanding of the molecular regulation underlying STB formation and function [36].

Regulation of trophoblast fusion. STB formation is driven by the transcription factor GCM1, which induces expression of fusogenic proteins Syncytin-1 and Syncytin-2, mediating CTB fusion and syncytial integrity. A recent experimental study using trophoblast organoids and primary culture models identified TFEB as an important regulator of syncytiotrophoblast differentiation and endocrine competence. During syncytialization, TFEB translocates to the nucleus, binds to promoters of fusogenic genes (Syncytin-1, Syncytin-2) and steroidogenic genes (e.g., CYP19A1), thereby coordinating trophoblast fusion and hormone secretion. Functional experiments showed that TFEB deficiency impaired syncytium formation and endocrine output, while its restoration rescued these processes [37]. Cutting-edge single-cell RNA sequencing and trophoblast organoid models have dissected STB heterogeneity, spatial organization, and transcriptional programs. Organoid models reveal STB-specific marker expression profiles and facilitate in vitro studies of syncytialization, contributing to functional validation of regulatory pathways [38]. For instance, trophoblast organoids and primary cultures have been used to validate TFEB as a regulator of syncytial fusion and endocrine competence; while enabling mechanistic inference, these systems still lack maternal immune components and uterine hemodynamics [37,38].

STB ensures the maternal–fetal interface’s barrier function, produces hormones (e.g., hCG, human placental lactogen (hPL), CRH, placental growth factors), and releases extracellular vesicles and peptides into the maternal circulation that modulate maternal physiology. STB acts as an endocrine hub essential for metabolic adaptation, fetal development, and immune tolerance. Recent data suggest RSV-related ubiquitination pathways also modulate trophoblast differentiation and endocrine function [39].

Dysregulated STB markers and hormone expression are linked to idiopathic stillbirths and disorders like preeclampsia and fetal growth restriction—for example, RNA-seq comparisons show downregulation of STB-specific genes (PSG, CSH families, KISS1, CRH) in idiopathic stillbirth cases. This finding underscores the clinical relevance of STB integrity for pregnancy health [40].

### 2.5. Transition from Histiotrophic to Hemotrophic Nutrition

During the first trimester, embryonic growth is sustained predominantly by histiotrophic nutrition, i.e., uptake of uterine gland secretions (glycoproteins, lipids, growth factors) delivered to the chorionic surface and internalized by trophoblast and yolk-sac–derived pathways under low oxygen tension. Contemporary reviews reaffirm that this arrangement protects the conceptus from oxidative stress during organogenesis and is maintained by trophoblastic plugs that restrict arterial inflow into the intervillous space. In parallel, a placental–endometrial dialogue (including glandular stimulation by trophoblast/placental lactogens) sustains gland activity and histiotroph output [41,42].

A programmed hemodynamic switch ensues around 10–12 weeks’ gestation, when endovascular trophoblast plugs progressively dislodge, allowing maternal arterial blood to enter and percolate through the intervillous space—marking the onset of hemotrophic nutrition. This transition raises local oxygen tension, drives villous angiogenesis and capillary expansion, and coincides with transcriptional shifts in trophoblast lineages captured by single-cell and spatial atlases of the first-trimester maternal–fetal interface. Failure to achieve a timely, orderly transition perturbs placental redox balance and is implicated in downstream placental malperfusion phenotypes [43].

Morphologically and functionally, the switch is accompanied by rapid villous remodeling: (i) progressive thinning of the trophoblast covering, (ii) expansion and peripheralization of fetal capillaries, and (iii) formation of vasculosyncytial membranes that minimize diffusion distance for gas and nutrient exchange. These structural adaptations, together with the recruitment of 80–100 spiral arteries to supply low-resistance flow, consolidate the hemochorial architecture characteristic of the human placenta. Notably, the two modes of nutrition are not strictly mutually exclusive; a period of overlap is expected as perfusion matures regionally across the placental disc [42,43].

At a molecular level, the transition integrates oxygen-sensing pathways (HIF axis), angiogenic signaling (VEGF/PlGF), and endometrial-trophoblast cross-talk that modulates glandular secretion and vascular adaptation. Recent syntheses emphasize that the timing and magnitude of oxygen rise, together with trophoblast responses, are central to establishing a stable maternal perfusion domain and to preventing oxidative injury as the placenta shifts to hemotrophic supply [42].

## 3. Pathological Pathways in Placental Disease

Deficiencies in placental development lie at the heart of the major obstetrical syndromes—such as preeclampsia (PE), fetal growth restriction (FGR), placenta accreta spectrum (PAS), recurrent miscarriage, and complications specific to monochorionic twin pregnancies like twin-to-twin transfusion syndrome (TTTS). These conditions arise not from a single causative lesion but from the breakdown of complex and tightly regulated molecular and cellular processes that orchestrate trophoblast differentiation, villous morphogenesis, trophoblast invasion, and maternal–fetal vascular remodeling. Recent studies using multi-omic approaches and innovative model systems have highlighted several convergent pathogenic pathways shared across these disorders. Unbalanced hypoxia-inducible factor (HIF) signaling disrupts trophoblast proliferation and angiogenesis, while an imbalance between pro-angiogenic and anti-angiogenic factors—specifically elevated soluble Flt-1 (sFlt-1) and reduced placental growth factor (PlGF)—leads to maternal endothelial dysfunction [44,45].

In addition, immune-mediated maladaptation, particularly involving maternal uterine natural killer (uNK) cell receptor KIR and fetal HLA-C interactions, has been implicated in impaired trophoblast invasion and the occurrence of preeclampsia [46]. In PAS, persistence of an unnecessarily invasive trophoblastic phenotype enables excessive myometrial infiltration, while in monochorionic twin gestations, abnormal vascular anastomoses create hemodynamic imbalances responsible for conditions such as TTTS.

Pathological hallmarks such as accelerated villous maturation, increased syncytial knotting, fibrinoid necrosis, and aberrant vascular morphologies visually reflect these molecular disruptions and underscore the importance of connecting mechanistic insights to morphologic evidence. Understanding how these molecular and histological changes intersect is critical for the development of early diagnostic tools and tailored therapeutic strategies in placental pathology.

### 3.1. Preeclampsia and Fetal Growth Restriction

Impaired placental development, especially shallow trophoblast invasion and inadequate spiral artery remodeling, can play an important role in obstetrical syndromes including preeclampsia (PE) and fetal growth restriction (FGR). Such failures of placental development can lead to maternal hypertension and impaired fetal growth. However, it should be noted that FGR may also result from maternal comorbidities (such as diabetes or hypertension), infections, or lifestyle factors (e.g., smoking, alcohol use, and nutritional deficiencies). This failure leads to maternal vascular malperfusion and a cascade of hypoxia-reperfusion injury at the maternal–fetal interface. Recent multicenter cohort studies have confirmed that the imbalance between pro-angiogenic placental growth factor (PlGF) and anti-angiogenic soluble fms-like tyrosine kinase-1 (sFlt-1) is a hallmark of these disorders. In particular, the sFlt-1/PlGF ratio has shown very high negative predictive value for short-term risk stratification: in a large Japanese prospective study, a cut-off of 38 yielded an NPV of >98% for ruling out preeclampsia within one week of testing [47,48]. Meta-analyses have further validated that the ratio outperforms either marker alone in detecting disease onset [49]. Its clinical utility extends beyond prediction; high ratios correlate with severe maternal and fetal complications, underscoring its role as both a diagnostic and prognostic tool [50,51].

Clinically, defective adaptation of the oxygen-sensing pathway disrupts angiogenesis and predisposes to preeclampsia and FGR. The resulting oxidative stress damages villous structures and further exacerbates the angiogenic imbalance. Immune maladaptation at the maternal–fetal interface, particularly involving altered KIR/HLA-C combinations, impairs vascular remodeling and contributes to preeclampsia [52,53].

Clinically, the placenta in PE and FGR often exhibits histopathological features such as accelerated villous maturation, abundant syncytial knots, distal villous hypoplasia, and fibrinoid necrosis—all markers of ischemic and oxidative damage at the villous tree. These morphological changes reflect the underlying molecular derangements and provide a visual confirmation of placental dysfunction that aligns with circulating biomarker profiles.

**Figure 2:**
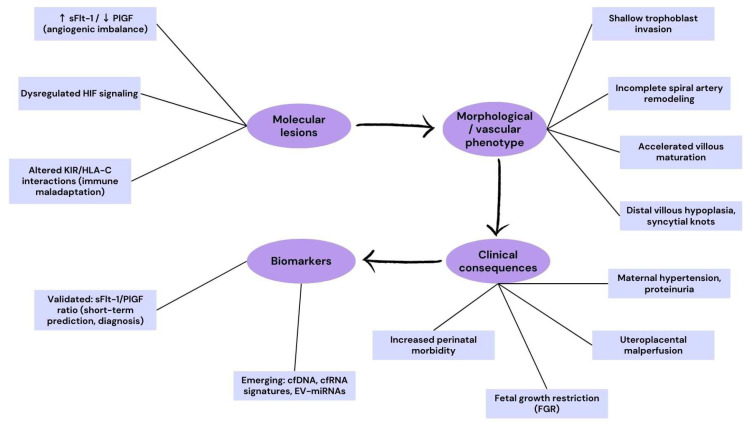
Translational pathway in preeclampsia and fetal growth restriction (PE/FGR). The flowchart maps the cascade from molecular lesions (↑ sFlt-1/↓ PlGF angiogenic imbalance, dysregulated HIF signaling, altered KIR/HLA-C interactions) to morphological/vascular phenotypes (shallow trophoblast invasion, incomplete spiral artery remodeling, accelerated villous maturation, distal villous hypoplasia, syncytial knots), leading to clinical consequences (maternal hypertension, proteinuria, uteroplacental malperfusion, fetal growth restriction, increased perinatal morbidity). The diagram also highlights biomarkers currently validated in clinical practice (sFlt-1/PlGF ratio) and emerging ones (cell-free DNA [cfDNA], cell-free RNA [cfRNA], extracellular vesicle–derived microRNAs [EV-miRNAs]).

To better illustrate the translational cascade from molecular mechanisms to clinical outcomes, we have included a schematic flowchart (Figure 2). This diagram integrates the major molecular lesions identified in preeclampsia and fetal growth restriction, their corresponding villous and vascular phenotypes, clinical consequences, and currently validated or emerging biomarkers. Such a representation reinforces the “molecular-to-morphology-to-clinic” continuum, providing a didactic tool for readers and trainees.

### 3.2. Placenta Accreta Spectrum (PAS)

Placenta accreta spectrum, encompassing accreta, increta, and percreta, is characterized by abnormal trophoblastic invasion that extends beyond the decidua basalis into the myometrium. Risks increase with the number of prior cesarean deliveries; in population-based cohorts, the risk of PAS reaches ~6.74% among women with ≥5 previous cesareans, whereas tertiary referral series may report higher figures due to referral bias [54,55]. Antenatal ultrasound is the first-line diagnostic modality for suspected PAS, with pooled sensitivity approaching ~90% and specificity ~97%. MRI serves as a complementary tool—particularly in posterior placentation or equivocal sonographic findings—by refining anatomical mapping and preoperative planning [54].

Fundamentally, PAS arises when scar tissue, often from prior cesarean sections or other uterine surgeries, compromises the integrity of the decidua basalis and the Nitabuch’s layer, creating a pathological “niche” that lacks the inhibitory signals normally restraining trophoblast invasion. Transcriptomic and mechanobiological investigations have confirmed that cesarean scars alter the biomechanical and biochemical properties of the uterine wall, leading to defective decidualization and over-invasive trophoblast behavior [56,57]. This loss of a decidual “stop signal” allows anchoring villi to breach the myometrial interface and, in severe cases, invade neighboring organs. 

On a molecular level, PAS exhibits a sustained invasive trophoblast phenotype typified by elevated levels of proteases, especially matrix metalloproteinase-9 (MMP-9), and reduced expression of epithelial markers such as E-cadherin. Immunohistochemical studies show that MMP-9 is significantly upregulated in PAS placental and decidual tissues compared to controls, supporting a proteolytic mechanism that fosters excessive tissue invasion [58,59]. Moreover, PAS has been linked to dysregulated non-coding RNA networks; for instance, altered expression of placenta-specific microRNA clusters such as C19MC and C14MC, along with regulatory pathways involving NF-κB and PTEN, have been identified in PAS tissue and may contribute to aberrant cell proliferation and invasion [60].

Clinically, PAS remains challenging to detect antenatally, and many cases are identified only at the time of delivery—delayed diagnosis substantially increases maternal and fetal morbidity. Beyond imaging, exploratory plasma proteomic signatures and circulating miRNAs have been reported; however, these candidates remain in the discovery/validation phase and are not yet ready for clinical implementation [61,62]. Quantitative proteomic studies have begun to explore potential early biomarkers for PAS; novel plasma proteins have been identified that may enable improved antenatal screening, though validation in larger cohorts is still required [61,62].

In summary, PAS exemplifies how anatomical disruption of the maternal decidual environment—especially by prior surgical scars—combined with dysregulated molecular trophoblast programming, can drive invasive placentation. The severity of PAS mandates accurate antenatal detection and delivery planning in appropriate care settings, highlighting the need for integrated imaging, molecular insight, and histopathologic correlation for improved maternal–fetal outcomes.

**Figure 3:**
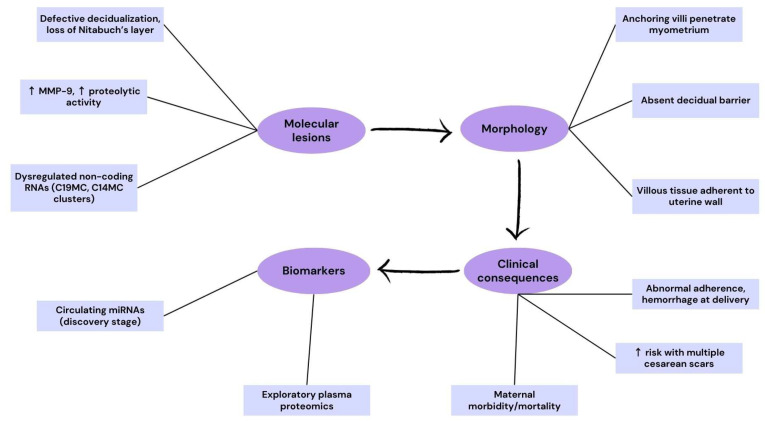
Translational pathway in placenta accreta spectrum (PAS). The schematic illustrates the cascade from molecular lesions (defective decidualization with loss of Nitabuch’s layer, ↑ MMP-9 and proteolytic activity, dysregulated non-coding RNAs [C19MC, C14MC clusters]) to morphological changes (anchoring villi penetrate the myometrium, absent decidual barrier, villous tissue adherent to the uterine wall). These abnormalities lead to clinical consequences (abnormal adherence, hemorrhage at delivery, increased risk with multiple cesarean scars, maternal morbidity/mortality). The flowchart also includes exploratory biomarkers under investigation (circulating miRNAs at the discovery stage, exploratory plasma proteomics).

To highlight the mechanisms underlying placenta accreta spectrum, we have included a schematic flowchart (Figure 3). This diagram integrates molecular alterations, abnormal placental morphology, clinical consequences, and exploratory biomarkers. It emphasizes how impaired decidualization and aberrant proteolytic activity translate into pathological adherence and increased maternal morbidity, reinforcing the continuum from molecular lesions to clinical manifestations.

### 3.3. Multiple Gestations and Monochorionic Complications

Monochorionic twin gestations, which arise when a single embryo splits after the chorion has formed, inherently share a single placenta and thus provide a unique milieu where placental vascular interdependence can give rise to severe complications. One of the most clinically significant conditions is twin-to-twin transfusion syndrome (TTTS), which affects approximately 10–15% of monochorionic twin pregnancies. TTTS is characterized by an imbalanced transfusion of blood via placental arteriovenous anastomoses, resulting in one hypovolemic donor twin and one hypervolemic recipient twin, leading to oligohydramnios in the former and polyhydramnios in the latter [63]. At a molecular level, TTTS placentas show evidence of hypoxia, oxidative stress, and ischemia–reperfusion injury, with emerging data implicating the involvement of VEGF signaling pathways and the renin–angiotensin system in the pathophysiology of the donor and recipient territories [63]. Clinical management has advanced through the use of fetoscopic laser photocoagulation, which ablates the vascular anastomoses and significantly improves survival rates; however, despite this intervention, residual oxidative damage and developmental compromise in the surviving twin may persist [64].

Recent biomarker research highlights plasma microRNAs as promising non-invasive diagnostic tools. For example, a study published in 2025 identified specific microRNAs in maternal plasma that were differentially expressed in pregnancies complicated by severe TTTS and exhibited strong predictive capacity for disease onset [65]. Given the high risk of TTTS, updated surveillance protocols have been proposed. The North American Fetal Therapy Network (NAFTNet), in a 2025 consensus, recommended enhanced ultrasound surveillance in monochorionic twins for early detection of both TTTS and the related twin anemia–polycythemia sequence (TAPS), underscoring the need for timely intervention [66]. Another relevant complication is selective intrauterine growth restriction (sIUGR), which can develop independently from TTTS and results from unequal placental sharing; meta-analyses of monochorionic twins point to discordant placental territories and vascular distribution as key contributors to differential nutrient exchange and fetal growth [66].

In summary, monochorionic placentation confers inherent risks due to vascular interconnections and unequal placental sharing. Conditions like TTTS and sIUGR reflect molecular and hemodynamic imbalances unique to this gestational type. Advances in molecular diagnostics, notably microRNA profiling, and enhanced ultrasound surveillance protocols now offer the potential for earlier detection and intervention, though long-term outcomes remain influenced by the timing and severity of these vascular insults.

**Figure 4:**
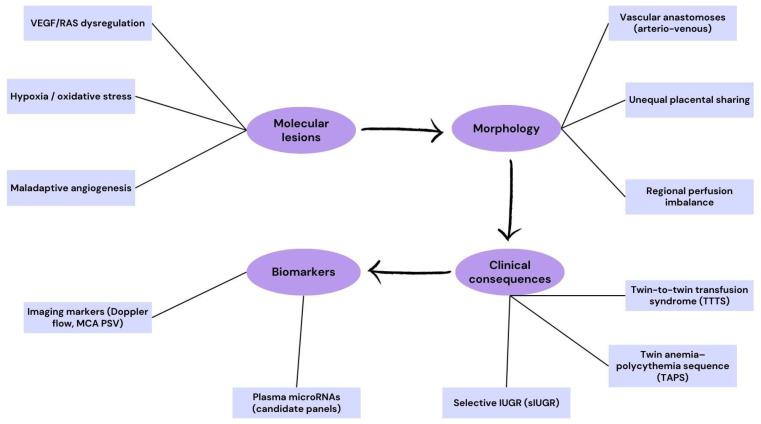
Translational pathway in monochorionic complications. The flowchart shows the progression from molecular lesions (VEGF/RAS dysregulation, hypoxia/oxidative stress, maladaptive angiogenesis) to morphological features (vascular anastomoses, unequal placental sharing, regional perfusion imbalance). These changes lead to clinical consequences such as twin-to-twin transfusion syndrome (TTTS), twin anemia-polycythemia sequence (TAPS), and selective intrauterine growth restriction (sIUGR). Biomarkers include imaging markers (Doppler flow, middle cerebral artery peak systolic velocity [MCA PSV]) and candidate plasma microRNA panels under evaluation.

To emphasize the cascade of pathogenic mechanisms in monochorionic pregnancies, we have included a schematic flowchart (Figure 4). This diagram illustrates how molecular dysregulation drives placental vascular abnormalities, which in turn lead to clinically significant complications such as TTTS, TAPS, and selective IUGR. The integration of biomarkers, including imaging parameters and plasma microRNAs, highlights the translational potential of mechanistic insights in guiding early diagnosis and monitoring.

### 3.4. Pregnancy Loss and Early Implantation Failure

Early pregnancy loss is increasingly recognized as the consequence of disrupted molecular pathways regulating implantation and early placental development. A critical determinant is the establishment of a physiologically hypoxic environment during the first trimester, maintained by trophoblastic plugs that occlude spiral arteries. Premature removal of these plugs leads to early maternal blood entry into the intervillous space, causing oxidative stress, mitochondrial dysfunction, and villous degeneration, ultimately predisposing to miscarriage [23].

Immune maladaptation at the maternal–fetal interface also contributes significantly. Extravillous trophoblasts normally express the non-classical MHC molecule HLA-G, which interacts with uterine NK cells to induce immune tolerance. Reduced HLA-G expression or unfavorable polymorphisms impair this dialogue, leading to excessive immune activation and recurrent implantation failure [67].

Cytokine signaling within the decidua is another key pathway. Leukemia inhibitory factor (LIF) and interleukin-11 (IL-11) are critical for decidualization and stromal remodeling, and insufficient expression disrupts trophoblast invasion. Single-cell transcriptomic studies have confirmed that downregulation of LIF and IL-11 in endometrial stromal cells directly correlates with defective implantation and early pregnancy loss [68].

Circulating microRNAs, particularly those packaged in placental exosomes, are increasingly studied for their potential as non-invasive biomarkers for early placental dysfunction. Placental miRNA clusters such as C19MC have been detected at high levels in maternal blood during early gestation and are enriched in exosomal fractions, suggesting a role in materno-fetal signaling (see Subramanian et al., 2023; Ghafourian et al., 2022). However, specific associations between C19MC downregulation and miscarriage remain to be validated in future large-scale longitudinal studies [69,70].

Histopathological findings in early pregnancy loss, including immature mesenchymal villi, poor vascularization, hydropic degeneration, and increased Hofbauer cells, mirror these molecular derangements. Thus, oxidative stress from premature perfusion, defective HLA-G mediated tolerance, inadequate cytokine signaling, and altered miRNA profiles converge to define the molecular pathology of early implantation failure.

**Figure 5:**
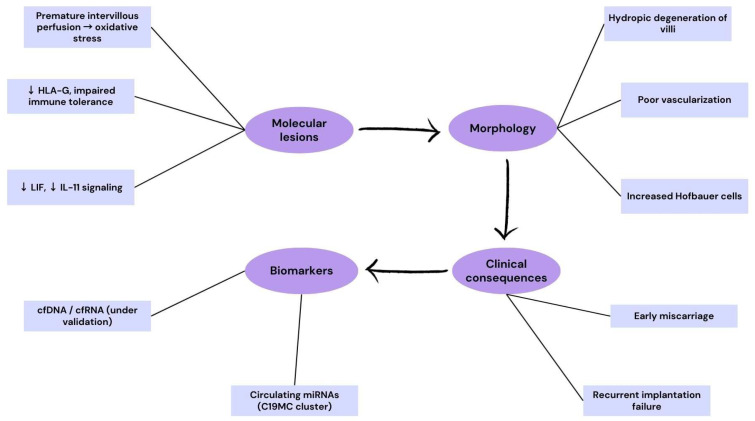
Translational pathway in pregnancy loss and early implantation failure. The flowchart depicts the continuum from molecular lesions (premature intervillous perfusion and oxidative stress, ↓ HLA-G with impaired immune tolerance, ↓ LIF and ↓ IL-11 signaling) to morphological changes (hydropic degeneration of villi, poor vascularization, increased Hofbauer cells). These abnormalities culminate in clinical consequences such as early miscarriage and recurrent implantation failure. Biomarkers under evaluation include circulating cell-free DNA/RNA (cfDNA, cfRNA), and microRNAs from the C19MC cluster.

To illustrate the mechanisms implicated in early pregnancy loss and implantation failure, we have included a schematic flowchart (Figure 5). This figure highlights how immune dysregulation, impaired cytokine signaling, and premature oxidative stress drive villous alterations, ultimately leading to miscarriage or recurrent implantation failure. The diagram also integrates candidate biomarkers that may support early diagnosis and risk stratification.

### 3.5. Structural and Vascular Variants

Structural placental variants—including velamentous or marginal cord insertion, bilobed or succenturiate lobes, and circumvallate morphology—reflect not only macroscopic abnormalities but also underlying molecular disruptions in villous development, angiogenesis, and trophoblast invasion.

Velamentous cord insertion (VCI) has been associated with aberrant angiogenic signaling. Abnormalities in the VEGF-PlGF axis, which regulate early chorionic vascular patterning, may predispose to misplaced cord insertion and unprotected vessel trajectories. Increased circulating sFlt-1 levels and reduced PlGF have been observed in pregnancies complicated by abnormal cord insertion, suggesting that anti-angiogenic imbalance contributes to impaired umbilical cord anchoring and placental vascular remodeling [71].

Bilobed and succenturiate placentas likely arise from regional defects in villous branching morphogenesis and chorionic plate expansion. Single-cell and spatial transcriptomic studies have demonstrated that villous patterning depends on tightly regulated Notch and Wnt signaling, as well as stromal-trophoblast interactions that guide angiogenesis. Perturbation of these developmental cues may result in lobulated placental architecture, with vulnerable interlobar vessels more prone to rupture [18].

Circumvallate placenta is linked to impaired trophoblast-decidual interactions at the placental margins. Insufficient EVT invasion and localized decidual hypoxia contribute to thickened, rolled membranes and restricted exchange area. Local immune maladaptation, particularly reduced HLA-G expression, may exacerbate abnormal placental attachment [31].

Collectively, these morphological anomalies underscore that structural variants are not merely gross anatomical curiosities but manifestations of dysregulated molecular pathways in angiogenesis, villous morphogenesis, and maternal–fetal immune tolerance. This mechanistic perspective highlights their importance as both diagnostic markers and windows into the biology of placental development.

### 3.6. Molecular Crossroads of Placental Pathology

Despite the diversity of clinical manifestations, placental pathologies such as preeclampsia, fetal growth restriction, placenta accreta spectrum, pregnancy loss, and monochorionic twin complications share several converging molecular pathways. A central shared pathway is the oxygen-sensing network, where inappropriate HIF activity contributes across disorders from early miscarriage to preeclampsia [32].

Another major axis is the imbalance of angiogenic factors, particularly elevated levels of the anti-angiogenic soluble fms-like tyrosine kinase-1 (sFlt-1) and decreased placental growth factor (PlGF). This anti-angiogenic shift reduces maternal endothelial integrity, restricts placental blood flow, and underlies both preeclampsia and fetal growth restriction. The diagnostic and prognostic role of the sFlt-1/PlGF ratio has been validated in large multicenter cohorts, confirming its central role in stratifying risk for maternal and perinatal complications [47,49]. Immune maladaptation, through defective trophoblast–uNK signaling, is a recurring mechanism across placental disorders [30,31].

The process of syncytialization, driven by GCM1 and fusogenic retroviral proteins such as Syncytin-1 and Syncytin-2, represents another molecular hub. Inadequate syncytiotrophoblast formation impairs endocrine signaling, increases oxidative stress susceptibility, and reduces the efficiency of nutrient and gas exchange. Downregulation of syncytiotrophoblast markers, including PSG and CSH family genes, has been demonstrated in placentas from stillbirths, underscoring the importance of trophoblast fusion pathways in pregnancy outcomes [36].

In disorders such as placenta accreta spectrum, persistence of an invasive EVT phenotype is mediated by sustained activity of proteases such as MMP-2 and MMP-9 and deregulated non-coding RNA networks, leading to uncontrolled tissue infiltration. Recent molecular studies have confirmed upregulation of MMP-9 in PAS specimens, supporting the proteolytic model of excessive invasion [59].

Finally, in monochorionic twin pregnancies, aberrant vascular anastomoses reflect maladaptive angiogenesis and regional imbalances in placental vascular patterning. Multi-omic analyses suggest that altered VEGF signaling and endothelial stress underlie complications such as TTTS and TAPS [63].

Taken together, these converging pathways-oxygen sensing, angiogenic balance, immune regulation, trophoblast fusion, proteolytic invasion, and vascular patterning—define the molecular crossroads of placental pathology. Integrating these mechanistic insights provides not only a unifying framework for the great obstetrical syndromes but also a foundation for translational diagnostics and therapeutic strategies.

To synthesize these converging mechanisms, Table 1 summarizes the major molecular pathways implicated in placental disease, their key regulators, and the associated clinical phenotypes.

## 4. Diagnostics and Biomarkers

The early detection of placental dysfunction is critical for timely intervention and improved perinatal outcomes. Traditional diagnostic methods—such as ultrasound and maternal clinical assessments—often detect pathology only after clinical symptoms or structural changes have already occurred. In contrast, molecular biomarker profiling offers a transformative approach by revealing pathologic alterations in placental function before they translate into overt disease. Recent reviews affirm that comprehensive panels of molecular markers—including proteins, circulating nucleic acids, and extracellular vesicle cargo—have the potential to significantly enhance early risk stratification and precision medicine in obstetrics [72].

### 4.1. Angiogenic Markers (sFlt-1/PlGF Ratio)

The imbalance between angiogenic and anti-angiogenic factors is one of the most robustly validated molecular signatures of placental dysfunction. Soluble fms-like tyrosine kinase-1 (sFlt-1), an anti-angiogenic splice variant of the VEGF receptor, binds and neutralizes VEGF and placental growth factor (PlGF), thereby reducing their bioavailability. This shift toward an anti-angiogenic state impairs maternal endothelial function, disrupts uteroplacental circulation, and plays a central role in the pathogenesis of preeclampsia and fetal growth restriction.

The sFlt-1/PlGF ratio has emerged as a clinically actionable biomarker, with multiple prospective multicenter trials confirming its predictive and diagnostic utility. A 2025 multicenter Japanese cohort demonstrated that an elevated ratio accurately predicted the development of preeclampsia and related adverse outcomes, with a negative predictive value exceeding 98% for short-term risk assessment [48]. Similarly, meta-analyses published in 2025 confirmed that the ratio outperforms either marker alone in predicting both early- and late-onset disease [49].

Beyond prediction, the ratio has been integrated into clinical management algorithms. A 2023 guideline review in Ultrasound in Obstetrics & Gynecology concluded that sFlt-1/PlGF testing provides substantial value not only for diagnosis but also for monitoring disease progression and guiding delivery timing [50]. The translational impact is particularly evident in reducing unnecessary hospitalizations by reassuring clinicians when ratios remain below threshold.

Thus, the sFlt-1/PlGF ratio exemplifies how mechanistic insights into placental angiogenesis can be translated into routine obstetric practice. Its success also sets the precedent for the validation of emerging biomarker panels that integrate proteomic, transcriptomic, and extracellular vesicle data to further improve early diagnosis of placental dysfunction.

### 4.2. Placental Proteins (PAPP-A, PP13, hCG, hPL)

Several placenta-derived proteins have emerged as important translational biomarkers reflecting underlying placental pathology. Pregnancy-associated plasma protein A (PAPP-A), a metalloproteinase that modulates IGF bioavailability, has been consistently associated with adverse outcomes when measured at low maternal serum levels during the first trimester. Lower PAPP-A levels correlate significantly with increased risks of IUGR, preeclampsia, and gestational hypertension [73]. Additionally, a 2023 study confirmed that low first-trimester PAPP-A levels are linked to placental insufficiency and poor fetal growth trajectories [74]. When combined with biochemical markers such as PlGF, low PAPP-A enhances the prediction of adverse outcomes before 34 weeks gestation [75].

Placental Protein 13 (PP13), also known as galectin-13, is another candidate with demonstrated predictive value. In women at risk for preeclampsia, PP13 levels measured in the first trimester were significantly lower than in controls, suggesting a role in early identification of placental dysfunction [76]. Furthermore, a meta-analysis supports its utility in preeclampsia screening, though it highlights the need for larger prospective studies to validate clinical implementation [77]. More recent work indicates that PP13, along with ORAI1 and FGF23, shows altered expression in late-onset preeclampsia, reflecting involvement in calcium and phosphate signaling pathways [78].

hCG, specifically its beta subunit (hCGβ), reflects syncytiotrophoblast activity. Elevated placental and mitochondrial dysfunction–associated hCGβ expression has been documented in FGR and preeclampsia with FGR, possibly mediated by HIF1α stabilization and downstream inflammatory pathways [79]. These data suggest that hCG dysregulation may serve as a molecular indicator of placental metabolic stress.

Human placental lactogen (hPL), while less studied in recent literature, remains an established marker of placental endocrine function and reflects essential trophoblastic processes. Though contemporary molecular studies are limited, hPL’s role in maternal metabolic adaptation underscores its potential as a biomarker in contexts such as gestational diabetes and fetal growth anomalies.

### 4.3. Nucleic Acid–Based Biomarkers (cfDNA, miRNAs, lncRNAs)

Placental nucleic acids that enter the maternal circulation provide a direct molecular readout of trophoblast physiology and have emerged as powerful candidates for the early, non-invasive detection of placental dysfunction. Cell-free placental DNA (cfpDNA) is measurable throughout gestation and reflects placental (epi)genomic states, with mounting evidence that quantitative and qualitative cfDNA signals (e.g., fetal fraction, fragmentation, methylation) track placental health rather than fetal genotype alone [80]. Leveraging routinely collected cfDNA sequencing data, recent studies demonstrate that genome-wide features from standard prenatal screening can be repurposed to predict preeclampsia risk early in pregnancy (≤16 weeks), underscoring the translational potential of cfDNA as a placental biomarker beyond aneuploidy testing [81]. Complementary work with cell-free RNA (cfRNA) shows that disease-specific transcriptional programs are detectable well before clinical onset of preeclampsia, revealing stable early-gestation cfRNA signatures of endothelial, immune, and smooth muscle pathways that mirror placental pathophysiology [82]. Together, these findings position circulating nucleic acids as an integrated, systems-level window into placental biology, with cfDNA/cfRNA panels poised to augment or even precede angiogenic testing in risk stratification algorithms [83,84].

Circulating microRNAs (miRNAs)—released directly by trophoblast or exported within extracellular vesicles (EVs)—capture post-transcriptional control of invasion, angiogenesis, syncytialization, and immune crosstalk at the maternal–fetal interface, and multiple first-trimester miRNAs show consistent association with later placental complications in systematic and narrative syntheses [69,85]. Targeted discovery studies further indicate that specific maternal-plasma miRNAs (e.g., miR-194-5p, miR-1278) can enhance early prediction of preeclampsia when combined with established clinical and biochemical markers, supporting their utility in multimodal screening frameworks [86].

Because most placenta-derived miRNAs circulate in EVs (exosomes/microvesicles) that protect and traffic functional cargo, EV-miRNA signatures are increasingly favored for robustness and biological specificity; recent reviews and cohort analyses highlight EV-encoded miRNAs as informative readouts of trophoblast stress, endothelial dysfunction, and inflammatory signaling in preeclampsia, FGR, gestational diabetes mellitus, and preterm birth [87,88,89].

These EV-based strategies align with mechanistic data showing that placental exosomes increase across gestation and actively modulate maternal vascular, metabolic, and immune responses, thereby encoding disease-relevant molecular perturbations that can be measured in blood long before overt clinical signs appear [70]. Long non-coding RNAs (lncRNAs) add another regulatory layer with diagnostic promise. Placental lncRNA networks are altered in preeclampsia and related placental disorders, and emerging placental and circulating data point to hierarchical lncRNA control of trophoblast migration, angiogenesis, and oxidative-stress responses that underlie early-onset disease biology [90,91].

As analytic pipelines mature, multi-analyte assays integrating cfDNA/cfRNA with EV-miRNA and lncRNA signals are likely to yield high-dimensional biomarker panels that capture the polygenic, multicellular nature of placental pathology and improve early risk stratification beyond single-marker approaches [80,87].

### 4.4. Extracellular Vesicles and Exosome Signatures

EVs, particularly exosomes secreted by the syncytiotrophoblast into the maternal circulation, have emerged as key mediators of intercellular communication at the maternal–fetal interface. These vesicles contain proteins, lipids, DNA, and regulatory RNAs (including microRNAs and lncRNAs), thereby reflecting the physiological and pathological state of the placenta. Quantitative studies demonstrate that circulating EVs increase progressively with gestational age, while abnormal concentrations or altered cargo composition are consistently reported in pregnancies complicated by preeclampsia, fetal growth restriction, gestational diabetes, and preterm birth [87].

Molecular profiling of exosomal miRNAs has proven especially informative. In preeclampsia, specific exosome-packaged miRNAs, including members of the placenta-specific C19MC cluster, are dysregulated, correlating with endothelial dysfunction, oxidative stress, and immune imbalance. Such EV-miRNA signatures are increasingly proposed as robust biomarkers, since vesicular encapsulation protects nucleic acids from degradation, ensuring stability in maternal plasma [88]. Moreover, comparative analyses show that EV-derived miRNAs outperform total circulating miRNAs in distinguishing between normal and pathological pregnancies, underlining their diagnostic superiority [89].

Proteomic studies of placental EVs have further identified cargo proteins linked to angiogenic imbalance, immune modulation, and metabolic regulation. For example, exosomes from preeclamptic placentas exhibit increased levels of anti-angiogenic factors, reinforcing their mechanistic role in the sFlt-1/PlGF disequilibrium that drives disease pathogenesis [92]. In addition, functional studies demonstrate that placental exosomes can actively alter endothelial cell function, vascular tone, and maternal immune responses, supporting the concept that they are not only biomarkers but also active effectors of disease biology [70].

Collectively, extracellular vesicles represent a promising next-generation biomarker class, offering a window into the dynamic molecular cross-talk between placenta and mother. Their integration into multimodal diagnostic platforms, alongside angiogenic factors and nucleic acid markers, could significantly enhance early prediction and personalized management of placental pathologies.

### 4.5. Multi-Omics Integration

The complexity of placental dysfunction cannot be fully captured by single biomarkers, as these syndromes arise from the convergence of multiple disrupted pathways. Multi-omics strategies—integrating genomic, transcriptomic, epigenomic, proteomic, and metabolomic data—are increasingly employed to construct composite biomarker panels that reflect the multifactorial nature of placental pathology. Recent work has shown that single-cell and spatial multi-omics provide unprecedented resolution of trophoblast subtypes, endothelial networks, and maternal immune cells, uncovering regulatory circuits that are altered in preeclampsia and fetal growth restriction [18].

At the transcriptomic level, multi-omic analyses combining cfRNA with proteomic signatures in maternal plasma can identify high-risk pregnancies months before clinical onset. A landmark study demonstrated that cfRNA signatures of placental and vascular origin are detectable in early pregnancy and can accurately predict later development of preeclampsia [82]. Similarly, integrative proteomic and metabolomic profiling has highlighted disease-specific pathways, such as altered angiogenic signaling and disrupted energy metabolism, which differentiate healthy from pathological placentas [27].

The translational potential of multi-omics lies in building composite diagnostic models. For example, combining angiogenic markers (sFlt-1/PlGF) with cfRNA or EV-miRNA signatures has been shown to substantially improve early prediction of preeclampsia compared with any marker alone [83]. Integration of epigenomic signals, such as placental DNA methylation, further adds predictive power and may reveal long-term fetal programming effects associated with abnormal placentation [80].

Together, these data suggest that multi-omic biomarker panels represent the future of placental diagnostics. By capturing the interplay of angiogenesis, immune regulation, trophoblast invasion, and metabolic pathways, multi-omics provides a holistic molecular fingerprint of placental health. The challenge remains to standardize assays and validate findings in large, diverse cohorts, but the integration of multi-omics into clinical pipelines holds promise to transform pregnancy care toward precision obstetrics.

A structured overview of current biomarker classes, their molecular basis, and clinical status is presented in Table 2.

## 5. Discussion and Future Directions

This review has highlighted how placental development is orchestrated by tightly regulated molecular pathways and how their dysregulation leads to the great obstetrical syndromes. A key message emerging from the synthesis of recent data is that common pathogenic nodes—including oxygen sensing, angiogenic balance, immune tolerance, and trophoblast differentiation—are repeatedly implicated across clinically distinct disorders such as preeclampsia, fetal growth restriction, placenta accreta spectrum, pregnancy loss, and monochorionic twin complications. This convergence suggests that diagnostic and therapeutic strategies targeting shared molecular pathways may have wide applicability across obstetric syndromes.

Despite significant advances, several knowledge gaps remain. First, while HIF and angiogenic signaling are well characterized in preeclampsia and growth restriction, the timing and dynamics of their disruption in early gestation are not fully understood. Second, although immune maladaptation through KIR/HLA-C and HLA-G interactions is strongly implicated in abnormal placentation, variability across populations and pregnancy phenotypes limits direct clinical translation. Third, while multi-omic and spatial atlases have begun to map cellular and molecular interactions at the maternal–fetal interface, integration of these high-dimensional datasets into clinically useful tools is still at an early stage. Finally, although biomarkers such as the sFlt-1/PlGF ratio have entered clinical practice, most candidate molecules—including PP13, miRNAs, exosomal cargo, and lncRNAs—require rigorous validation in large, ethnically diverse cohorts before they can be adopted.

Looking forward, the field is moving toward precision obstetrics, where risk prediction and patient management are guided by molecular signatures rather than clinical symptoms alone. Integration of multi-omic biomarker panels, including cfDNA/cfRNA, EV-packaged miRNAs, and proteomic/metabolomic signatures, into prospective screening programs could enable earlier and more accurate identification of pregnancies at risk. Moreover, advances in trophoblast organoids, placenta-on-chip systems, and spatial transcriptomics will not only refine our understanding of pathophysiology but also provide preclinical platforms for therapeutic discovery. The development of targeted interventions, such as antioxidants modulating oxidative stress or immune modulators enhancing maternal tolerance, will depend on translating these molecular insights into actionable therapies.

In summary, the future of placental research lies in bridging molecular pathology with translational applications. By combining mechanistic discoveries with high-dimensional biomarker integration and advanced model systems, it is possible to move closer to a personalized, predictive, and preventive approach to obstetric care.

## 6. Conclusions

Placental health is central to pregnancy success, and its failure underlies the spectrum of major obstetric syndromes. Advances in molecular biology have revealed that dysregulated oxygen sensing, angiogenic imbalance, impaired immune tolerance, and defective trophoblast differentiation are shared mechanisms across clinically diverse conditions. The integration of angiogenic markers, placental proteins, nucleic acid signatures, and extracellular vesicle cargo into multimodal diagnostic platforms represents a transformative step toward early, non-invasive prediction of disease. Looking ahead, the combination of multi-omic technologies with advanced experimental models offers unprecedented opportunities to translate mechanistic discoveries into personalized obstetric care. By bridging molecular insights with clinical practice, it may finally be possible to improve pregnancy outcomes and reduce the global burden of placental disease.

## Figures and Tables

**Table 1:** Major molecular pathways implicated in placental pathology, their representative regulators, and associated clinical phenotypes. Abbreviations: HIF—hypoxia-inducible factor; ROS—reactive oxygen species; VEGF—vascular endothelial growth factor; PlGF—placental growth factor; sFlt-1—soluble fms-like tyrosine kinase-1; KIR—killer immunoglobulin-like receptor; HLA—human leukocyte antigen; GCM1—glial cells missing 1 transcription factor; PSG—pregnancy-specific glycoprotein; CSH—chorionic somatomammotropin gene family; MMP—matrix metalloproteinase; EVT—extravillous trophoblast; PAS—placenta accreta spectrum; TTTS—twin-to-twin transfusion syndrome; TAPS—twin anemia–polycythemia sequence; FGR—fetal growth restriction; PE—preeclampsia; sIUGR—selective intrauterine growth restriction.

Molecular Pathway	Key Regulators	Placental Pathology	Representative Findings
Oxygen sensing & hypoxia [23,32]	HIF-1α, ROS balance	Preeclampsia, FGR, miscarriage	Premature plug loss, oxidative stress
Angiogenic balance [48,50]	VEGF, PlGF, sFlt-1	Preeclampsia, FGR	sFlt-1/PlGF ratio ↑, villous malperfusion
Immune tolerance [31,53]	KIR/HLA-C, HLA-G	Preeclampsia, miscarriage	Shallow invasion, immune rejection
Syncytialization [36,40]	GCM1, Syncytin-1/2	Stillbirth, FGR, PE	↓ PSG, ↓ CSH family gene expression
Proteolytic invasion [59]	MMP-2, MMP-9, NF-κB	PAS	Persistent EVT invasion, myometrial infiltration
Vascular patterning [63]	VEGF, Notch, Wnt	TTTS, TAPS, sIUGR	Abnormal vascular anastomoses

**Table 2:** Principal biomarker classes for placental disease, their molecular basis, and clinical utility. Abbreviations: sFlt-1—soluble fms-like tyrosine kinase-1; PlGF—placental growth factor; PAPP-A—pregnancy-associated plasma protein A; PP13—placental protein 13; hCG—human chorionic gonadotropin; hPL—human placental lactogen; cfDNA—cell-free DNA; cfRNA—cell-free RNA; miRNA—microRNA; lncRNA—long non-coding RNA; EV—extracellular vesicle; scRNA-seq—single-cell RNA sequencing; PE—preeclampsia.

Biomarker Class	Examples	Molecular Insight	Clinical Application/Status	Gestational Window (Best Performance)	Sample Type	Typical Thresholds	Evidence Grade
Angiogenic factors [48,49,50]	sFlt-1, PlGF	Angiogenic imbalance	Validated in PE (prediction, monitoring, delivery timing)	20–37 weeks (second and third trimester)	Maternal plasma/serum	sFlt-1/PlGF ratio >38 (rule-in), <38 (rule-out within 1 week)	Guideline-endorsed; validated in large cohorts
Placental proteins [73,76,79]	PAPP-A, PP13, hCG, hPL	Trophoblast invasion, syncytial & endocrine fn	In screening; complementary with angiogenic testing	First trimester (11–14 weeks)	Maternal serum	Low PAPP-A <0.4 MoM; variable for PP13/hCG	Clinically used for screening; moderate evidence
Nucleic acids [80,82]	cfDNA, cfRNA, miRNAs, lncRNAs	Genetic/epigenetic placental signals	Emerging; early prediction of PE and related complications	First–second trimester (10–24 weeks)	Maternal plasma	No standardized thresholds	Exploratory; discovery to early validation
Extracellular vesicles [87,88]	Exosomal miRNAs, proteins	Trophoblast stress, immune/vascular signaling	Discovery phase; promising for early non-invasive diagnosis	First–second trimester	Maternal plasma/serum	No validated cut-offs	Discovery phase
Multi-omics panels [18,27,83]	scRNA-seq, proteomics, metabolomics	Integrated placental fingerprint	Preclinical; precision obstetrics development	Variable (first–third trimester, depending on platform)	Placental tissue, plasma	Research-dependent	Preclinical/experimental

## Data Availability

Data available on request.
